# The Orai Pore Opening Mechanism

**DOI:** 10.3390/ijms22020533

**Published:** 2021-01-07

**Authors:** Adéla Tiffner, Lena Maltan, Sarah Weiß, Isabella Derler

**Affiliations:** Institute of Biophysics, JKU Life Science Center, Johannes Kepler University Linz, A-4020 Linz, Austria; lena.maltan@jku.at (L.M.); sarah.weiss@jku.at (S.W.)

**Keywords:** STIM1, Orai1, CRAC channels, ion channel structure–function relationship

## Abstract

Cell survival and normal cell function require a highly coordinated and precise regulation of basal cytosolic Ca^2+^ concentrations. The primary source of Ca^2+^ entry into the cell is mediated by the Ca^2+^ release-activated Ca^2+^ (CRAC) channel. Its action is stimulated in response to internal Ca^2+^ store depletion. The fundamental constituents of CRAC channels are the Ca^2+^ sensor, stromal interaction molecule 1 (STIM1) anchored in the endoplasmic reticulum, and a highly Ca^2+^-selective pore-forming subunit Orai1 in the plasma membrane. The precise nature of the Orai1 pore opening is currently a topic of intensive research. This review describes how Orai1 gating checkpoints in the middle and cytosolic extended transmembrane regions act together in a concerted manner to ensure an opening-permissive Orai1 channel conformation. In this context, we highlight the effects of the currently known multitude of Orai1 mutations, which led to the identification of a series of gating checkpoints and the determination of their role in diverse steps of the Orai1 activation cascade. The synergistic action of these gating checkpoints maintains an intact pore geometry, settles STIM1 coupling, and governs pore opening. We describe the current knowledge on Orai1 channel gating mechanisms and summarize still open questions of the STIM1–Orai1 machinery.

## 1. Introduction

### 1.1. The Ca^2+^ Ion—A Versatile Second Messenger

Calcium (Ca^2+^) ions play an essential role in controlling different biological processes within the human body. As a versatile second messenger, Ca^2+^ ions are involved in the control of a wide range of essential biological processes, such as gene transcription, proliferation, apoptosis, migration, and exocytosis. To ensure the correct processing of these signaling pathways, sustained Ca^2+^ levels are of utmost importance for healthy cells. Abnormal cytosolic Ca^2+^ concentrations can lead to severe diseases such as immune deficiencies and cancer [1,2,3]. Among a huge diversity of Ca^2+^ ion channels, those involved in the so-called store-operated Ca^2+^ entry (SOCE) play a considerable role in a variety of cell types. They are activated upon Ca^2+^ store depletion of the endoplasmic reticulum (ER). The most prominent store-dependent Ca^2+^ entry pathway represents the Ca^2+^ release-activated Ca^2+^ (CRAC) channel. Anomalous cytosolic Ca^2+^ concentrations, caused by either gain- (GoF) or loss-of-function (LoF) point mutations in the CRAC channel components, can lead to severe diseases such as York and Stormorken syndrome and tubular aggregate myopathy [4], severe combined immunodeficiency, autoimmunity, ectodermal dysplasia, and muscular hypotonia [5,6,7,8].

### 1.2. Overview of the Activation Cascade of the CRAC Entry

**Figure 1:**
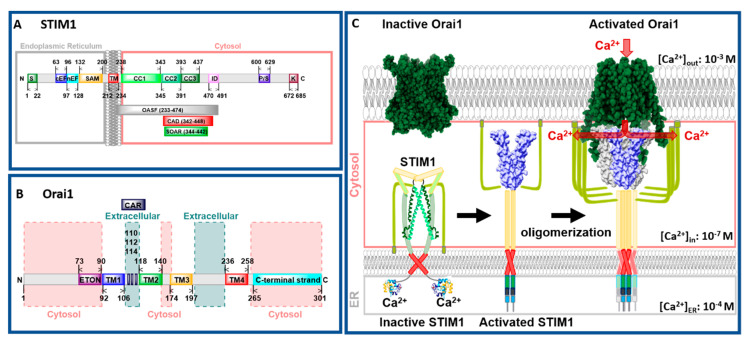
Stromal interaction molecule 1 (STIM1) and Orai1. (**A**) Scheme showing full-length human STIM1 with respect to regions critical for the regulation of the STIM1/Orai1 signaling cascade. Important minimal STIM1 C-terminal fragments, such as OASF (Orai1-activating small fragment), CAD (Ca^2+^ release-activated Ca^2+^ channel (CRAC) activation domain) and SOAR (STIM1–Orai1-activating region) are further represented as insets. (**B**) The scheme shows the full-length human Orai1 channel with highlighted regions and residues that are crucial for the Orai1 function. (**C**) The scheme visualizes STIM1 and Orai1 in the resting state *(left)* and STIM1 extended state established upon store depletion at ER–PM junctions *(middle)*. STIM1 punctae formation, that is achieved via oligomerization, is followed by STIM1 binding to the Orai1 C-terminus, which presumably extends ~45 Å into the cytosol *(right)*.

CRAC channels are composed of two molecular key components, the ER membrane-embedded stromal interaction molecule (STIM) that acts as the Ca^2+^ sensor and the pore-forming Orai protein located in the plasma membrane (Figure 1A,B). STIM proteins are single-span ER transmembrane (TM) proteins composed of the ER-luminal Ca^2+^ binding site, the so-called EF–sterile α-motif (SAM) complex, and a cytosolic C-terminal region containing three helical coiled-coil segments (CC1, CC2, and CC3) followed by a flexible region (Figure 1A). Orai channels form hexameric complexes. Each Orai subunit contains four TM domains connected via three loops and flanked by the cytosolic N- and C-terminus [7,9] (Figure 1B). There are two STIM protein isoforms (STIM1 and STIM2) and three Orai protein variations (Orai1–3) among which STIM1 and Orai1 are sufficient to constitute the CRAC channel [10,11].

CRAC channel activation is initiated via the binding of a ligand to its respective receptor in the plasma membrane (PM), which leads to the production of phospholipase C (PLC) in the cytosol. In turn, phosphatidylinositol 4,5-bisphosphate (PIP_2_) and subsequently inositol 1,4,5-trisphosphate (IP_3_) are generated. IP_3_ binds to the IP_3_ receptor located in the ER membrane and thereby releases Ca^2+^ from the ER into the cytosol. STIM1 proteins, assumed to occur as dimers under resting cell conditions [12,13,14], sense the drop in the Ca^2+^ concentration. Subsequently, they undergo a conformational change [15,16,17,18,19], oligomerize [15,18,19], and migrate to ER–PM junctions [15,18,20]. There, the cytosolic side of STIM1, in particular, two (CC2, CC3) of the three helical coiled-coil segments (Figure 1A,C), bind to and activate the Orai1 channel [9,10,19,20,21,22,23,24,25,26,27,28]. STIM1 binding to Orai1 leads to a global conformational change of the channel complex that results in an open conformation and accordingly in Ca^2+^ influx into the cell (Figure 1C). After refilling of the ER, STIM1 senses a higher Ca^2+^ concentration, unbinds from, and inactivates Orai1. Finally, STIM1 undergoes a conformational change back to its resting state. This CRAC channel activation mechanism is unique among Ca^2+^ ion channels [3,29,30,31,32,33]. 

Structural studies [34,35,36] complemented by a series of molecular dynamics (MD) simulations [37,38,39,40,41,42,43,44,45] revealed new detailed insights into the understanding of the STIM1 and Orai1 activation mechanism. So far, for STIM1, only the structures of N- and C-terminal STIM1 fragments are available [46,47,48,49,50]. The structure of full-length STIM1 is not yet resolved. It is predicted that the N-terminal Ca^2+^ binding site of STIM1 is connected to its TM domain. Subsequently, the C-terminal region, composed of three helical structures, is assumed to adopt a tight conformation in the quiescent state and an extended conformation in the STIM1 active state (Figure 1C).

Recent studies published four main crystal and two cryo-EM (cryogenic electron microscopy) structures of *Drosophila melanogaster* Orai (dOrai) [34,35,36,51], which are discussed in more detail later in the review. These structures consistently revealed a hexameric stoichiometry of the Orai channel and a single pore-forming region in the center of the complex. These structures allowed resolving inter- and intramolecular interactions within Orai and between Orai and STIM. Furthermore, these structural studies bring us one step closer to understanding and resolving the unique mechanism of CRAC channel activation. Structural resolutions of human Orai variants (sequence homology of 73% compared to dOrai [52]) are highly awaited.

Throughout the review, we refer to dOrai only when describing Orai structural resolutions, whereas functional effects are presented only for human Orai channels. For simplicity, we write e.g., Orai1 for the human Orai1 variant, rather than hOrai1. 

## 2. Functional and Structural Properties of Orai1 Channel Activation and Stoichiometric Requirements

### 2.1. Biophysical Features and Authentic Hallmarks of CRAC Channels

STIM1-induced Orai channel activation leads to strongly inward rectifying Ca^2+^ currents as known from endogenous CRAC channels [53]. Their typical biophysical features include high Ca^2+^ selectivity, small single-channel conductance, Ca^2+^-dependent feedback regulation, and enhancements in currents in a divalent free sodium (Na^+^) (I_DVF_)- versus a Ca^2+^ (I_Ca^2+^_)-containing solution [33,54], which are described below. 

CRAC channels represent the most Ca^2+^-selective ion channels with a permeability ratio of Ca^2+^ versus Na^+^ higher than 1000 [55,56]. Since their single channel conductance is only in the range of 10–30 fS [57], single-channel openings cannot be resolved. Strongly inward rectifying CRAC channel current/voltage relationships exhibit a reversal potential higher than +50 mV [58,59]. CRAC channels can conduct small monovalent ions (Na^+^, lithium (Li^+^), potassium (K^+^)) as long as the extracellular solution is free of divalent ions. The presence of Ca^2+^ ions within a concentration range of µM blocks monovalent Na^+^ currents, which represents the anomalous mole fraction behavior of Ca^2+^ over Na^+^ currents [60]. 

The high Ca^2+^ selectivity of CRAC channels is attained via the narrow pore diameter (3.8–3.9 Å) at the selectivity filter and the Ca^2+^-accumulating region (CAR) located in the first external loop of Orai1. The pore diameter of the Orai1 channel is significantly lower than that of voltage-gated Ca^2+^ (Ca_V_) channels. Indeed, cesium (Cs^+^) is hindered from permeating through the Orai1 channel in contrast to the L-type Ca_V_ and transient receptor potential vanilloid subfamily member 6 (TRPV6) Ca^2+^ ion channels [61,62]. Moreover, STIM1 binding to Orai1 is essential to maintain high Ca^2+^ selectivity, as revealed via electrophysiological studies on the constitutively active Orai1 V102A/C mutants. Both mutants are nonselective in the absence of STIM1, but selective upon STIM1 binding, underlining the dynamic aspect of the Ca^2+^ selectivity [63,64].

The inactivation of Orai channels is regulated via the so-called Ca^2+^-dependent inactivation (CDI) to prevent excessive Ca^2+^ entry into the cell. As summarized in detail in Krizova et al. [33], this essential feedback mechanism can be categorized into fast and slow CDI. The fast Ca^2+^-dependent inactivation (FCDI) occurs within milliseconds, while the slow Ca^2+^-dependent inactivation (SCDI) occurs on a timescale of several minutes. FCDI can be observed as a decrease in CRAC currents during a hyperpolarized voltage step [65]. It is determined by cytosolic regions in STIM1 and Orai1 [24,54,66] and the STIM1:Orai1 expression ratio [67,68]. The SCDI can be monitored within time-course experiments subsequent to the activation of maximum currents. The ER-located, single TM span, accessory protein: store-operated Ca^2+^ entry-associated regulatory factor (SARAF), has been reported to control SCDI [69,70].

The prominent enhancements of I_DVF_ over I_Ca^2+^_ require STIM1 coupling and an intact Orai1 N-terminus [64]. It has been assumed that the permeability ratio Na^+^ versus Ca^2+^ (I_DVF_:I_Ca^2+^_) correlates with the extents of CDI obtained in the respective solutions [53]. The abrogation of inactivation in a Na^+^-containing DVF solution is a possible explanation for a ratio I_DVF_:I_Ca^2+^_ being higher than 1. 

Summarizing, STIM1-mediated Orai1 channel currents exhibit several biophysical properties which make up typical CRAC channel currents. As outlined later in the review, we discovered that single-point mutations or other alterations within the Orai channel can lead to a change in the typical CRAC channel hallmarks. This suggests that certain structures involved in the maintenance of the well-known CRAC channel properties are lacking. 

### 2.2. Structural Features of Orai Proteins

**Figure 2:**
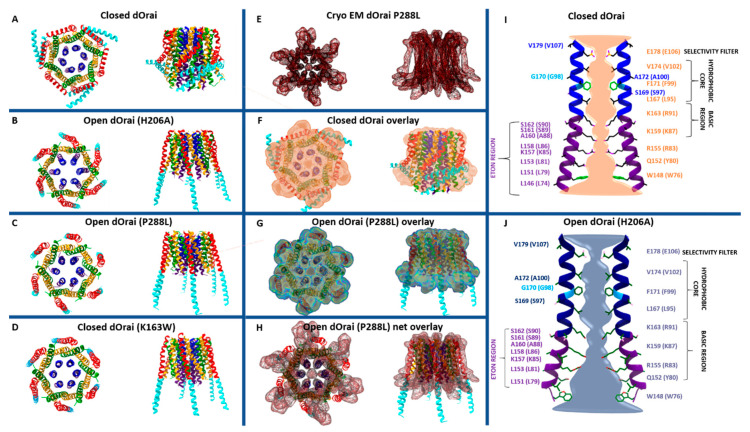
Structure of Orai channel. (A–D) Top and the corresponding side view of the crystal structure of the closed dOrai (**A**), the open state of dOrai H206A (analogue to Orai1 H134A) (**B**), the open state of dOrai P288L (analogue to Orai1P245L) (**C**), and the closed state of dOrai K163W (analogue to Orai1 R91W) (**D**) are shown. (**E**) Top and the corresponding side view of the net cryo-EM (cryogenic electron microscopy) structure of the open state of dOrai P288L (analogue to Orai1P245L). (**F**,**G**) Overlay of cryo EM dOrai P288L structure with the closed dOrai and open dOrai P288L crystal structures. (**H**) Overlay of net cryo EM dOrai P288L structure with open dOrai P288L crystal structure. (**I**,**J**) The pore of the closed dOrai channel and open dOrai H206A are displayed (in brackets, the corresponding position in Orai1 is stated). The Ca^2+^-accumulating region (CAR) region, selectivity filter, hydrophobic core, basic region, and extended transmembrane Orai1 N-terminal (ETON) region are visualized by their respective residues.

Among the currently available structures, the two closed-state structures include dOrai wildtype and a LoF mutant (dOrai K163W) (Figure 2A,D), while the open-state structures contain single-point mutations known to induce constitutive activity (H206A (in Orai1 H134A) or P288L (in Orai1 P245L)) (Figure 2B,C,E) independent of STIM1 [34,35,36,51]. Among the open-state structures, only the recent cryo-EM structure of dOrai H206A shows a high resolution with 3.3 Å [51]. 

Taking a closer look on the individual subunits, the four TM domains are connected by two extracellular (TM1/TM2 and TM3/TM4) and one intracellular (TM2/TM3) loop [7,9] and flanked by a cytosolic N- and C-terminus. It is noteworthy that each TM domain displays an extended helical segment reaching into the cytosol. The most outstanding ones represent that of TM1, the extended transmembrane Orai1 N-terminal (ETON) region, and that of TM4, the extended TM4 (TM4ext or C-terminus) (Figure 1B and Figure 2). Both cytosolic extensions are essential for STIM1-mediated Orai1 activation [35].

The pore is established by the six TM1 domains within the hexameric complex, which extend ~20 Å into the cytosol [35]. The cytosolic extension of TM1 forms a helical segment of the last third of the N-terminus, known as the ETON region [64]. The TM1-pore region is surrounded by a tightly packed ring formed by TM2 and TM3, which is crucial to maintain the communication between TM1 and TM4, indispensable for Orai1 channel function. The outer ring of the channel complex is formed by the last transmembrane region, TM4. Its helical structure is divided into two parts (TM4a, TM4b) by a kink (in Orai1 P245, in dOrai P288) roughly in the middle of the plasma membrane. At the cytosolic side of TM4b, a hinge region (Orai1 L261–K265), the so-called nexus region, connects TM4 with its helical extension TM4ext [35] (Figure 2). Among the Orai loop regions, only the linkage between TM1 and TM2 was resolved by the most recent cryo-EM structure of dOrai H206A (Orai1 H134A). These extracellular loop1 regions were described to shape structured turrets which are stabilized via a network of hydrophobic interactions. They establish an electronegative pore entrance [51] matching with the findings that the three glutamates in each loop1 function as a CAR [45].

An overall comparison of all available closed and open structures hint at potential conformational changes upon Orai pore opening, yet in the absence of STIM1. Main differences were detected within the pore region and the helical segments at the outmost side of the channel complex. Consistently, the pore region of all open structures exhibits a dilation of the basic region of approximately 10 Å (Figure 2I,J) compared to 6 Å in the closed structures [34,35,36,51,71]. The most drastic structural differences occur within the channel periphery. In the closed state, the C-termini of adjacent subunits cross each other in an antiparallel manner and form a belt-like structure around the channel due to coiled-coil interactions (Figure 2A). In the open crystal structures, the entire TM4 C-terminus segment is fully straightened toward the cytosol via unlatching conformational changes (dOrai H206A and P288L) (Figure 2B,C) [34,51]. Contrarily, the cryo-EM open (dOrai P288L) structure shows neither the antiparallel pairing nor a straightening of the TM4 helices, but rather a tight packing of TM3/TM4 (Figure 2E–J) [34]. The recent cyro-EM structure of dOrai H206A revealed an outward movement of each subunit, which amounts to approximately 5 Å at the cytosolic side of TM4 [51]. Interestingly, structural resolution of a LoF mutant dOrai K163W (analogue to Orai1 R91W) exhibits also a straightened TM4 C-terminus region, while the arrangement of other TM domains is comparable to that of the quiescent Orai state [35,36] (Figure 2D). These observations on an intermediate unlatched conformation suggest that structural alterations along the TM4 C-terminal region are not sufficient for pore opening. 

Furthermore, MD simulations revealed that Orai pore opening involves twist-to-open gating motions with counterclockwise rotations of all TM1 regions at the extracellular side and a dilation of the pore. At the intracellular side, gating movements occur in an alternate manner, with three subunits moving outward, while the other three subunits rotate in a clockwise manner [41]. Contrarily, the most recent cryo-EM dOrai H206A open structure revealed rigid body movements of each subunit, rather than a rotation of individual TM domains [51]. Noteworthy, all structures were obtained in the absence of STIM1. Thus, further studies are required to resolve so far unknown STIM1 triggered alterations of the structural features of Orai channels. 

Overall, while pore dilation upon Orai1 activation is well accepted, the rearrangements at the outmost side of the channel complex are still a big topic of discussion in the community. It still needs to be resolved why the crystal and the cryo-EM structures are fundamentally different (Figure 2F–H) and how the channel changes during activation in vivo. It is unclear whether the huge structural changes along TM4 and C-terminus are energetically favorable under physiological conditions. Furthermore, structural and functional studies are required to resolve functional relevant conformational changes at the Orai channel periphery. Moreover, the recent open structures with higher resolution [51] together with potential future resolutions will provide better insights into a structural reorientation of single residues upon the switch from a closed to an open state.

### 2.3. Crucial STIM1-Binding Sites within Orai1

ER depletion-induced STIM1–Orai1 coupling involves a series of critical sites within the cytosolic regions of both proteins. As this review is in particular focused on the Orai activation mechanisms, we specifically emphasize on the STIM1-binding sites within Orai1.

Within Orai1, especially the cytosolic regions, i.e., N-terminus, loop2 region, and C-terminus, are worth being considered as STIM1-coupling sites. Currently, there is indisputable evidence that the Orai1 C-terminus represents the main coupling site for STIM1. Indeed, biochemical studies revealed a strong interaction of the STIM1 C-terminal fragment (CRAC-activating domain (CAD)) region with the Orai1 C-terminus [22]. In particular, L273 and L276 in Orai1 C-terminus were found to play a profound role in the interaction with the STIM1 C-terminus. Their mutation to more hydrophilic residues (S/D) leads to an abrogation of STIM1 binding [26,72,73,74,75,76]. Residues R281, L286, and R289 are also involved in STIM1 coupling [77]. Moreover, manipulation of the nexus region (amino acids (aa) 261–265), which represents a kinked connection between TM4 and the C-terminus, via site-directed mutagenesis leads to interference with STIM1 coupling. In detail, the Orai1 _261_ANSGA_265_ mutant, causing constitutive activity, exhibits reduced STIM1 coupling [78]. This indicates, on the one hand, that a specific conformation of the Orai1 C-termini is required for STIM1 coupling. On the other hand, these studies suggest that STIM1 coupling to the Orai1 C-terminus induces conformational changes required for pore opening, which is in line with the currently available crystal structures [34,35,36]. 

In addition to the Orai1 C-terminus, the N-terminus and the loop2 region play an extensive role in STIM1–Orai1 coupling. Biochemical and Förster resonance energy transfer (FRET)-derived interaction in a restricted environment (FIRE) experiments revealed an interaction of the STIM1 C-terminal fragments with the Orai1 N-terminus [22,79] and loop2 [43]. It is worth mentioning that the interaction of STIM1 C-terminal fragments with the Orai1 C-terminus was found to be stronger than that with the Orai1 N-terminus [22,76,79,80]. However, as those experiments were performed with fragments, the results need to be verified in an alternative manner using longer fragments or, in the best case, full-length Orai1. 

Nevertheless, manipulation of the ETON region by either point mutations (e.g., Orai1 K85E) or deletions (Orai1 ΔN_1–76/78_) results in reduced STIM1 binding [64,73,74]. More importantly, the conserved portion of the N-terminus establishes authentic CRAC channel hallmarks by fine-tuning Orai1 channel gating [54]. However, it is still unknown if the Orai1 N-terminus is directly or indirectly involved in the communication with STIM1. 

Additionally, recent studies provide clear evidence for the involvement of loop2 in Orai1 gating. Butorac et al. [81] discovered a direct interplay between the α3 domain of STIM1 and Orai1 loop2. The α3 domain is a small helical region (aa 400–403) within the CAD/SOAR (STIM1–Orai1-activating region) domain of STIM1. The deletion of the STIM1 α3 domain led to abrogated Orai1 channel activation. We were able to show the proximity of STIM1 L402 and Orai1 E166 by performing cysteine crosslinking experiments, as well as enhanced Ca^2+^ currents after diamide application through functional studies. 

Furthermore, an interplay of the N-terminus and loop2 determines functional STIM1–Orai1 coupling. The exchange of Orai1 loop2 with that of Orai3 recovers the activation of some inactive Orai1 N-terminal deletion mutants, likely due to the release of an inhibitory interaction of the truncated N-terminus and the loop2 in Orai1 [43]. Analogue experiments on diverse GoF mutants showed that, upon N-truncation (aa 78 in Orai1, aa 53 in Orai3), constitutive activity is only maintained in the presence of the Orai3 loop2, but not that of Orai1. These effects occur already independently of STIM1 [43]. Interestingly, crosslinking of the N-terminal residue K78 with loop2 E166 nearly abrogates STIM1-induced CRAC channel currents. Additionally, we recently demonstrated [82] in accordance with Dong et al. [41] that two intra-subunit (R83–E149, K85–E173) and one inter-subunit (K85–E149) salt-bridge interaction formed between the N-terminus and the loop2 region are indispensable for intact CRAC channel activation. They are involved in the establishment of functional STIM1 coupling to Orai1, as the LoF Orai1 mutations K85E and E149K impaired STIM1 coupling. These findings are further supported by our GoF/LoF double-point-mutant approach [82], as explained in detail in Section 3 and Section 4. At least always two of those salt bridges are required to be intact to retain STIM1-mediated Orai1 activation. Overall, an opening permissive communication of the N-terminus and loop2 is required to maintain an intact STIM1/Orai1 communication [43]. The cytosolic loop region likely acts as a bridge for the communication with the Orai1 N-terminus and, thus, the pore [82].

Summarizing, the Orai1 C-terminus represents the main direct coupling site for STIM1 C-terminus and a vital requisite for the communication of STIM1 with the Orai1 N-terminus and loop2. While direct, functionally relevant STIM1-C-terminus–Orai1-loop2 coupling has been reported, it is still unclear whether the Orai1 N-terminus also functions as a direct interaction partner for the STIM1 C-terminus. It is also probable that the interplay of the Orai1 N-terminus and loop2 establish together a functional indispensable STIM1-binding pocket. At least several salt-bridge interactions seem to be involved in accomplishing a correct communication of the N-terminus, loop2, and STIM1 C-terminus. However, further studies are still required to map crucial STIM1-binding pockets within loop2 and the N-terminus. 

### 2.4. Stoichiometry of STIM1 for Orai1 Activation

Crystal structures of dOrai and concatemeric studies of Orai1 revealed a hexameric stoichiometry of the Orai channel complex [35,36,83]. This leads to the suggestion that, within one STIM1–Orai1 complex, six STIM1 molecules bind to one Orai1 channel. Currently, three different models of STIM1 coupling to Orai1 are proposed. Along with the 1:1 STIM1:Orai1 stoichiometry, NMR (nuclear magnetic resonance) studies suggest that a dimer of STIM1 C-terminal fragments binds to the antiparallel-oriented C-termini of Orai1, the so-called bimolecular binding model [77]. Alternatively, fluorescence recovery after photobleaching (FRAP) and super-resolution microscopy studies imply a global 1:1 STIM1:Orai1 stoichiometry, with each C-terminal strand of the STIM1 dimer binding to an Orai1 subunit of two neighboring Orai1 channels. Indeed, the expression of Orai1 and SOAR fragments showed induced clustering of Orai1 channels, whereas SOAR mutations (e.g., STIM1 F394H) failed to induce Orai1 channel clustering. This way of STIM1/Orai1 interaction has been termed the unimolecular binding model [28]. Another alternative to the above-described models is the sequential step model. Several studies showed that a ratio of 2:1 of STIM1:Orai1 is required for maximal Orai1 activation, implying that 12 STIM1 proteins bind to the six subunits of Orai1 [67,68,84,85,86]. Palty et al. [86] suggested that initially one strand of the STIM1 dimer binds to one Orai1 subunit, inducing conformational changes within Orai1, leading to a slightly open state. Via the latter, the affinity for STIM1 binding is increased. Finally, the second strand of the STIM1 dimer binds to the same Orai1 subunit, fully activating the Orai1 channel. 

In addition to these models, Yen and Lewis [87] showed that an L273D substitution in Orai1, which prevents STIM1 binding, within one out of six C-termini of an Orai1 concatemer, leads to a strongly reduced open probability (~90%). This indicates a nonlinear dependence of Orai1 gating and STIM1 binding. This point mutation also caused weaker Ca^2+^-binding affinity, lower Na^+^ selectivity over Cs^+^, and increased single-channel conductance. Overall, their results underline the necessity of STIM1 binding to all six subunits of the Orai1 channel complex to ensure effective CRAC channel gating, Ca^2+^ selectivity, and low single-channel conductance. However, the exact stoichiometry of the STIM1–Orai1 complex is still elusive. Additional structural and functional studies are required to clarify the stoichiometry of the STIM1–Orai1 complex and whether the distinct stoichiometries regulate different cellular signaling pathways. 

## 3. Dynamics within the Orai Channel Complex upon Pore Opening 

### 3.1. The Orai1 Pore

The high Ca^2+^ selectivity of CRAC channels [55] arises from a sophisticated synergy of several segments within the pore-forming TM1 domains (Figure 2I,J). These include, from the extracellular to the intracellular side, the CAR, the selectivity filter, a hydrophobic cavity, and a basic region.

Within Orai1, Ca^2+^ ions are attracted by three negatively charged residues (D110/D112/D114) in the CAR [45]. Mutation of one of these residues decreased Ca^2+^ permeation in line with a shift in the density profile of Ca^2+^ ions calculated via MD simulations [45]. The CAR is integrated into a turret region extending 20 Å above the selectivity filter [51]. Functional studies together with MD simulations further revealed that salt-bridge interactions of the two extracellular loops of Orai channels compete with Ca^2+^ binding to the CAR region [45].

Subsequently, Ca^2+^ ions pass the selectivity filter (E106), the narrowest part of the pore. Interestingly, a comparison of the closed dOrai with the recent open cryo-EM dOrai H206A structure revealed that the side chains of the glutamates show distinct orientations. While, in the closed state, all are oriented downward within the pore, in the open structure, they exhibit alternate upward and downward orientations [51].

The selectivity filter is followed by a hydrophobic gate made up of the pore-lining residues V102, F99, and L95. Mutation of the positions V102, F99, or G98 can result in constitutive Orai1 currents in the absence of STIM1 [88,89,90], for instance, for V102A/C/G/S/T or F99Y/M/S/T/W/C/G. In line, MD simulations revealed that the valine-to-alanine substitution enhances the hydration of the pore compared to the wildtype Orai channel [42]. Interestingly, while, in the absence of STIM1, the currents of most of the constitutive mutants were nonselective, co-expression of STIM1 restored the Ca^2+^ selectivity of the respective mutants [39,88,89]. Water-soluble thiol reagents such as Cd^2+^ enabled discovering a rotation of residues in the hydrophobic cavity, due to their ability to form stable Cd^2+^ bridges between water-accessible cysteine residues located in close proximity. Examination of cysteine mutants (Orai1 G98C, Orai1 F99C) for Cd^2+^ permeation provided clear evidence that Orai1 pore opening most probably involves a rotation of G98 into the pore, while F99 moves away. Consequently, the energy barrier for ion permeation is lowered and G98C becomes accessible to the divalent metal ions in the Orai1 open state. The proposed rotation within the hydrophobic segment of TM1 is predicted to dynamically govern the high Ca^2+^ selectivity of Orai channels [88]. These experimental findings that G98 rather than F99 is exposed to the open pore are in line with MD simulations [88]. Moreover, the recent cryo-EM dOrai H206A structure [51] also resolved a rotation of this hydrophobic segment. Furthermore, the pore opening is accompanied by a widening of the hydrophobic region by more than 2 Å [51]. 

Additionally, the Ca^2+^ selectivity of Orai1 channels is assumed to be controlled via residues in TM3 (E190, K198), as demonstrated via functional and MD simulation studies [38,91]. 

The cytosolic segment of the Orai1 pore region consists of several basic residues (R91, K87, and R83) (Figure 2I,J). Frischauf et al. [44] suggested that pore opening involves a rotation of R91 toward S90 of the neighboring subunit. Others proposed that anions are attracted by the positively charged residues to either function as a plug, thus keeping the channel in a closed state [35], or help Ca^2+^ to pass the channel via cation–anion interactions in the open state [34,42,51]. A recent study by Yamashita et al. [37] suggested that neither the anion plug nor the proposed rotation of R91 toward S90 within the basic region is essential for Orai1′s pore opening. They suppose that the basic residues in the inner pore possess rather a long-range effect, maintaining hydration of the outer pore region. Indeed, point mutations by residues with neutral side chains abolished STIM1-mediated Orai1 activation and reduced pore hydration. The open dOrai structures showed a dramatic widening of the basic region, which is thought to be essential for the passing of Ca^2+^ ions into the cell [34,36,51] (Figure 2J). The high resolution of the cryo-EM dOrai H206A mutant revealed further that the side chains along the basic region are not rotated compared to the closed structure. Nevertheless, they are less defined than other pore-lining residues suggesting that they are more flexible [51]. Thus, it still remains to be determined whether STIM1 enables inducing a reorientation of the Orai1 N-terminus/TM1 region, as specifically proposed for the functional relevant R83–E149 salt-bridge interaction [41,82].

In summary, current experimental and structural studies suggest that Ca^2+^ permeation is initiated by the attraction of Ca^2+^ ions to the CAR region and their coordination at the selectivity filter. Ca^2+^ permeation is accomplished by a rotation of F99 out and G98 into the pore and by pore dilation along the TM1 domains, with the largest widening within the basic region. Despite G98 and F99 being supposed to alter their orientation during pore opening, the rest of TM1 is suggested to undergo no rotation on the basis of recent structural resolutions of dOrai constitutively active mutants [51].

### 3.2. Orai Gating Necessitates Several Checkpoint Residues in All TM Domains to Be Intact

**Figure 3:**
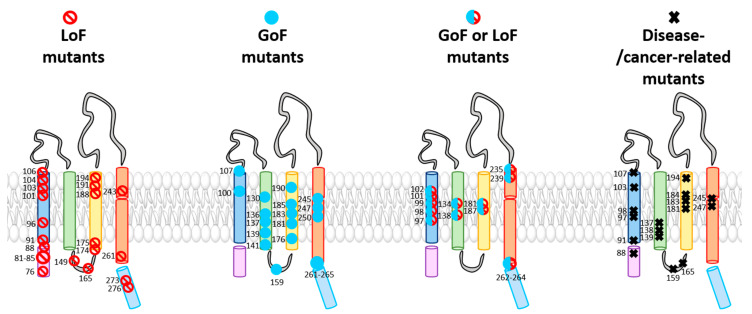
Orai1 mutations. The subunit schemes display so far discovered positions that, upon single substitution, lead to either LoF (indicated by red stop sign, *left*) or GoF (visualized by blue circle, *middle left*) mutations. The positions that, upon substitution to different residues, can lead to both GoF, i.e., Orai1 H134A, and LoF, i.e., Orai1 H134W, are represented (*middle right*). Disease-/cancer-related mutation positions are marked as black crosses (*right*).

**Figure 4:**
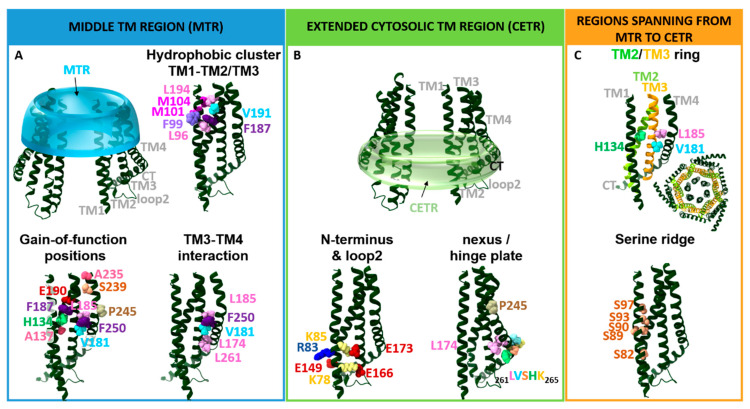
Important gating checkpoints. (**A**) The scheme highlights the location of the MTR region (cyan) (*top left*, **A**). The Orai1 subunit depicts the residues within the TM1–TM2/TM3 hydrophobic cluster (*top right*, **A**). The Orai1 monomer displays positions of the residues within the MTR that lead to GoF upon the single-point mutation (*bottom left*, **A**). Residues that mediate the TM3–TM4 interaction are visualized (*bottom right*, **A**). (**B**) The scheme highlighting the location of the CETR region (green) (*top middle*, **B**). The Orai1 monomer depicts the residues responsible for the communication between the Orai1 N-terminus and loop2 (*bottom left*, **B**). Orai1 results in the constitutively active channel upon fourfold mutation of the C-terminal kink at 261LVSHK265 to 261ANSGA265 and single-point mutation of the TM4 kink at P245 to any other amino acid, suggesting that these regions mediate the closed state of the channel (*bottom right*, **B**). (**C**) The Orai1 top view highlights the TM2/TM3 ring which spans from MTR to CETR. The monomer displays the crucial gating checkpoint residues of the TM2/TM3 ring. The serine ridge seems to provide flexibility within the conformation of the inner pore and is assumed to be responsible for the communication with the polar surfaces of TM2/TM3 ring (*bottom middle*, **C**).

The TM domains of the Orai1 complex include a multitude of checkpoints controlling Orai1 pore opening and/or STIM1 coupling (Table 1). Interestingly, these gating loci are not only located within the pore-lining TM1, but also in all other TM domains surrounding the pore. The evidence for these hotspots originates either from Orai1 mutants found to be responsible for CRAC channel-related diseases or functional evaluation of diverse Orai1 mutants designed to study the CRAC channel structure–function relationship (Figure 3 and Table 1). Two independent screens of TM residues via site-directed mutagenesis uncovered more than a dozen of GoF mutants [30,82]. We recently demonstrated that the series of checkpoints can be classified on the basis of their functional roles and location into two groups: those located in the middle transmembrane region (MTR) and those located in the cytosolic extended transmembrane region (CETR) (Table 1 and Figure 4). Gating loci in the MTR maintain the closed state and some of those additionally control an opening permissive conformation. Gating hotspots in the CETR are essential for STIM1 coupling and maintenance of an opening permissive conformation. These checkpoints are explained in detail in Section 4. Here, we aim to highlight some general features of these sites required for an intact Orai1 complex.

**Table 1:** Crucial Orai1 gating checkpoints. Summary of residues in Orai1 functioning as crucial gating checkpoints together with their location within Orai1, known gain- (GoF) and loss-of-function (LoF) mutations at the respective positions, and their relevance in disease (N-term., N-terminus; C-term., C-terminus).

Orai1	Location	GoF	LoF/*LoF^weak^*	Disease	Reference
**R83**	N-term. (CETR)		R83A		[41,82]
**K85**	N-term. (CETR)		K85E		[41,82]
**S89**	N-term. (CETR)				[92]
**S90**	N-term. (CETR)				[92]
**R91**	TM1 (MTR)		R91W (dOrai K163W)	Immunodeficiencies	[7,92]
**S93**	TM1 (MTR)				[39]
**L96**	TM1 (MTR)				[39]
**S97**	TM1 (MTR)	S97C		Stormorken-like syndrome	[39,93]
		S97M/L/I/V			[39]
**G98**	TM1 (MTR)	G98S		Tubular aggregate myopathy	[94,95]
			G98R	Immunodeficiencies	[96]
**F99**	TM1 (MTR)	F99Y/M/S/T/W/C/G			[88]
**M101**	TM1 (MTR)	M101F			[39,89,97]
**V102**	TM1 (MTR)	V102A/C/G/S/T	V102D/W		[63]
**A103**	TM1 (MTR)		A103E	Immunodeficiencies	[98]
**M104**	TM1 (MTR)				[39]
**V107**	TM1 (MTR)	V107M		Tubular aggregate myopathy	[99]
**H134**	TM2 (MTR)	H134A/C/S/T (dOrai H206A)	H134W		[36,39,44,82]
**F136**	TM2 (MTR)	F136A/S			[82]
**A137**	TM2 (MTR)	A137V		Colorectal tumor	[44,82]
**L138**	TM2 (MTR)	L138F		Tubular aggregate myopathy	[44]
**M139**	TM2 (MTR)	M139V		Stomach carcinoma	[44]
**S141**	TM2 (MTR)	S141C			[39]
**T142**	TM2 (MTR)		*T142C*		[82]
**E149**	loop2 (CETR)		E149K/R		[41,82]
**S159**	loop2 (CETR)	S159L			[44]
**E166**	loop2 (CETR)				[43,81]
**E173**	TM3 (CETR)		E173K		[41,81,82]
**L174**	TM3 (CETR)		L174D/K		[78,82]
**A175**	TM3 (CETR)		A175D/K		[78]
**W176**		W176A/C/S			[54,82,100]
**V181**	TM3 (MTR)	V181A/C/S			[54,82]
			V181SfsX8	Autoimmunity, ectodermal dysplasia	[96]
**G183**			G183D	Glioblastoma	[44]
**T184**	TM3 (MTR)	T184M		Tubular aggregate myopathy	[99]
**L185**	TM3 (MTR)	L185A (Orai3 F160A), L185C/S			[54]
**F187**	TM3 (MTR)	F187A/C/S			[34,39,82]
**L188**	TM3 (MTR)		*L188S*		[82]
**E190**	TM3 (MTR)		E160Q		[101]
**V191**	TM3 (MTR)				[39,82]
**L194**	TM3 (MTR)		*L194S/N*		[39,82]
			L194P	Autoimmunity, ectodermal dysplasia	[96]
**A235**	TM4 (MTR)	A235C	A235W		[39,82]
**S239**	TM4 (MTR)	S239C	S239W		[39,82]
**M243**	TM4 (MTR)		*M243S*		[82]
**P245**	TM4 (MTR)	P245X (X = any canonical aa); (dOrai P288L),		Stormorken-like syndrome	[5,34,39,82,102]
**G247**	TM4 (MTR)	G247S			[44]
**F250**	TM4 (MTR)	F250A/C/S			[39,54,82]
**L261**	TM4 (CETR)	L261A/C/S	L261D/K		[78,82]
**261LVSHK265**	TM4 ext	ANSGA			[78]
**V262**	TM4 ext	V262N			[82]
**L273**	C-term.		L273D/S		[26,72,74,85,102]
**L276**	C-term.		L276D/S		[26,72,74,85,102]

Among LoF mutants also LoF^weak^ mutants are included (highlighted in italic and underlined. While LoF mutations impair STIM1-mediated activation and act dominant over GoF mutations, LoF^weak^ mutations only impair STIM1-mediated activation (for more details see Section 3.3)). For known structures, corresponding positions in dOrai are mentioned. TM, transmembrane; CETR, cytosolic extended transmembrane region; MTR, middle transmembrane region; aa, amino acid.

With respect to the entire library of known Orai1 mutations (Figure 3 and Table 1), remarkably, all so far identified GoF mutants are widely located within the central segments of the TM regions and in a helical segment of TM4 close to the extracellular side, namely, the MTR (Figure 4) [82]. It seems that these particular residues act together allosterically within this conical region of the channel complex to control the closed and open Orai channel states. 

Among the currently known GoF mutants in the MTR, most of them contain a mutation from a strongly hydrophobic amino acid to a cysteine, serine, or alanine [39,43,44,54]. This suggests that typically small residues with low hydrophobicity can induce at certain sites a conformational change from the closed to the open state. However, at some positions, the constitutive activity could be obtained via substitution from a small to a larger hydrophobic amino acid (e.g., Orai1 M101F [97], Orai1 A137V, and Orai1 L138F [44,95]). Hence, whether a single-point mutation can induce GoF depends a lot on its location within the channel complex. 

Strikingly, it has been shown for some gating checkpoints in the MTR that, instead of a mutation leading to a GoF mutant, substitution to a residue with strongly different features in hydrophobicity and/or size can cause LoF [39,44,63,82,88,103] (Figure 3 and Table 1). Thus, a single gating hotspot within an Orai1 TM domain can control both pore opening and the maintenance of an opening-permissive Orai1 conformation. This is known for instance for the GoF mutants V102C/A, Orai1 H134A, and Orai1 L138F and their corresponding LoF mutants Orai1 V102D/W [63], Orai1 H134W, and Orai1 L138A [44]. Interestingly, the substitution of P245 at the kink within TM4 to any of the 20 canonical amino acids leads to GoF. These examples highlight again that no general dependence of LoF and the properties of the respective inserted residue at diverse gating checkpoints exists, but rather that the location of the amino acid determines whether a mutation leads to GoF or LoF.

Additionally, a variety of other LoF mutants resolved a set of additional checkpoints crucial for Orai1 function (Figure 3, Figure 4 and Table 1). Essential gating regions represent, in the MTR, the hydrophobic cluster at the interface of the TM1 and TM2/TM3 ring [39] and, in the CETR, the cytosolic triangles [82] and the hinge plate [28,78] (Figure 4), which are explained in detail in Section 4. For those positions, no substitutions are currently known that lead to GoF [39,82]. It remains to be determined whether a mutation of such residues can lead to constitutive activity. 

Most LoF mutants located in the MTR do not interfere with STIM1 coupling, indicating that they impair signal propagation to the pore or affect the pore geometry. LoF mutants in the extended cytosolic helical portions impair both opening-permissive channel and pore conformation and STIM1 coupling (Figure 4). 

In addition to the discovery of an arsenal of GoF and LoF mutations (Figure 3 and Table 1), it is also essential to obtain a detailed molecular understanding of the role of individual gating checkpoints. These essential gating loci likely interplay or form interactions with surrounding residues to maintain the closed state, which upon activation probably break to form novel interactions to stabilize an open state. Among all known gating loci, the role of H134 in TM2, as well as of a novel sulfur–aromatic interaction within Orai1, is well characterized [39,44,97], as described in detail in Section 4. Nevertheless, it is still outstanding and crucial for an enhanced understanding of Orai1 activation to determine how other positions lock the channel in the closed state, control an opening-permissive pore conformation, and/or establish functional STIM1 coupling. No general correlations between GoF or LoF and the property of the amino acid inserted at a certain gating locus exist [82]. Hence, it is of interest which amino-acid properties are required at a certain gating locus either to induce GoF and/or LoF or to maintain a conformation only opening-permissive for STIM1-mediated activation. 

### 3.3. Global Conformational Changes within the Orai Complex Are Indispensable for Pore Opening

Despite the stoichiometry of the STIM1–Orai1 complex not yet being clear, it is reasonable to assume that each Orai1 subunit couples to at least one STIM1 protein. Thus, STIM1 coupling to each Orai1 subunit in the channel complex initiates a gating signal that is transmitted to the pore. 

One great question in the CRAC channel field is how the STIM1-induced activation signals are relayed from the Orai1 C-termini of each subunit at the channel periphery to the pore in the center of the complex. The series of currently reported Orai1 GoF and LoF mutants led to the hypothesis that collective, interdependent motions of TM domains establish STIM1-induced pore opening [39,43,44,54,82,88,94,99,102]. The rigid body of the TM2/TM3 ring [30], as well as sulfur–aromatic interactions of adjacent subunits [97], has been recently reported to enforce signal transmission from the C-terminus to the pore and, thus, collective TM domain motions. Moreover, the recent cryo-EM structure revealed that pore dilation is established via rigid body outward movements of each subunit by 5 Å [51]. 

Our recent finding provided clear evidence that global conformational changes are indispensable for Orai1 pore opening [82]. For that, we recently generated a library of Orai1 double-point mutants, each combining one GoF and one LoF mutation in a variety of combinations [82] (Table 2). Thus, double-point mutants contained either the GoF or the LoF mutation in a TM domain closer to the pore compared to the LoF or GoF mutation, respectively. Some double-point mutants contained the GoF and the LoF mutation in the same TM domain. Independent of the location of these point mutations relative to each other and to the pore, the LoF mutation acts in a dominant manner over most GoF mutations (Table 2). Altogether, this demonstrates that the clearance of a series of gating checkpoints and a global conformational change of all TM domains are necessary for pore opening [82] (Table 3). Some LoF/GoF combinations led even within a dimer to LoF [82] (Table 3), which highlights that cooperativity of Orai1 subunits plays a significant role in pore opening [30,31]. 

**Figure 5:**
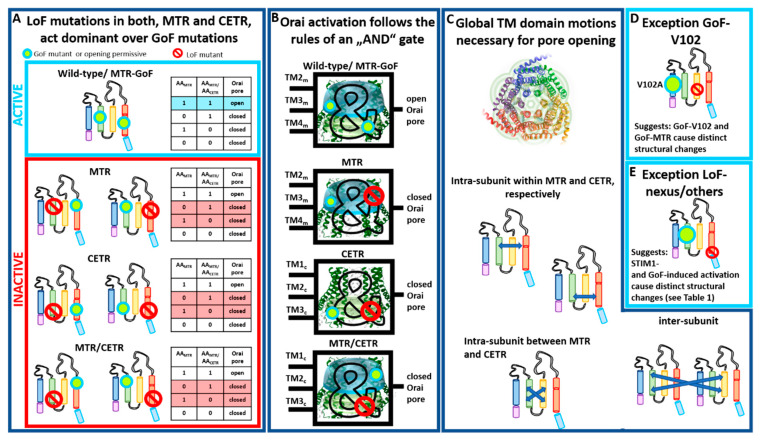
Global conformational changes in the Orai complex are indispensable for pore opening. A series of gating checkpoints need to capture an opening-permissive conformation to achieve an activation of Orai pore. Our library of double mutants [82] always revealed a dominant effect of the LoF mutation. (**A**) This suggests that, as soon as one of the multiple checkpoints in the Orai channels is defective (not in an opening-permissive conformation), the Orai channel cannot be activated. This behavior can be compared or even described with the rules of the Boolean relations. They are typically used in digital electronics, and one of those relations is the AND gate. The AND gate is an operator that connects two or more inputs with one output. As long as all inputs are 1, the output is also 1. As soon as one of the inputs is 0, then the output is also 0. These relations can be nicely used to describe the dependence of Orai1 gating checkpoints with Orai1 pore opening (individual tables in (**B**)). Only as long as all checkpoints in the MTR are 1 can the pore open. This holds also for the CETR. Thus, both MTR and CETR can be viewed as two individual AND gates. (**B**) Nevertheless, they can also be combined and, overall, all inputs (checkpoints) from MTR and CETR together can be viewed as an AND gate (**B**). (**C**) Global motions leading to Orai1 pore opening can be visualized as spherically propagating waves with the activation signal either from a constitutively active mutation or from STIM1 coupling to the Orai1 C-terminus (C, *top*). Not only intra-subunit interactions within MTR and CETR, but also between MTR and CETR from two different planes impact each subunit (**C**, *middle, bottom*). Additional inter-subunit interactions across membrane planes (MTR and CETR) of adjacent subunits influence the entire Orai1 channel complex. Within our screen, two exceptions were discovered (GoF V102 and LoF nexus), whose behavior is visualized and described in the right top (**D**) and right middle panel (**E**).

Interestingly, these dependencies can be described with the truth table of an AND gate, typically used in digital electronics (Figure 5A,B) [82]. Figure 5A,B illustrate the principle of the AND gate with always two gating checkpoints within an Orai subunit, whether both in the MTR, both in the CETR, or one in the MTR and one in the CETR. Only as long as both checkpoints adopt an opening permissive conformation can the Orai1 channel open. As soon as one checkpoint captures a nonpermissive conformation, the pore cannot open anymore (Figure 5B). This principle not only holds for two gating checkpoints in comparison, but can be extended to all known gating checkpoints. As soon as one of all those hotspots adopts a nonpermissive conformation, the Orai channel cannot open. We explicitly showed this behavior for Orai1 double mutants containing the LoF and GoF mutations, whether both in the MTR, one in the MTR and one in the CETR, or one in the first and one in the second subunit of a dimer [82]. Thus, we can conclude that pore opening requires concerted TM domain motions within each subunit and between adjacent subunits (Figure 5C).

Noteworthy, a few of the double mutants containing one LoF and one GoF mutation retained constitutive activity (Table 2). One of those exceptions represents the prominent GoF Orai1 mutation V102A in TM1 (Figure 5D and Table 2), which acts dominant over any LoF in the MTR or CETR [82], except R91W [104]. In accordance with these observations, it behaves also distinctly with respect to the authentic CRAC channel hallmarks [33,54] compared to a series of other known GoF mutants. Thus, Orai1 V102A likely induces divergent conformational changes within the channel complex, or pore opening is solely established via pore dilation. Interestingly, a recent report discovered another Orai1 TM1 mutation M101F, already very selective in the absence of STIM1, which also acted dominant over the LoF mutation H134W [97]. Here, further investigations are still necessary. 

Moreover, in contrast to a series of LoF mutations in the MTR and CETR, a few mutations, termed MTR LoF^weak^ (T142C, L188S, L194S/N, M243S) (Table 1, Table 2 and Table 3), lead only to LoF upon activation via STIM1, but retain GoF when combined with a GoF point mutation [82] (Table 2). This suggests variations in the activation pathways for STIM1-mediated Orai1 and GoF mutation-induced activation. Indeed, the biophysical characteristics of CRAC channel and GoF mutant currents are slightly distinct [32,33,54] (Figure 5E). The structural determinants responsible for these variations still need to be resolved, potentially via the comparison of highly awaited high-resolution structures of an Orai1 GoF mutant channel and, in particular, a STIM1–Orai1 channel complex.

Moreover, our LoF/GoF double mutants allowed distinguishing whether certain gating checkpoints are only involved in establishing an opening-permissive pore geometry or also in STIM1 coupling (Table 3). As described in Section 2, especially residues in the loop2 region connecting TM2 and TM3 (E149, L174) control both pore opening/hydration and STIM1 coupling. A crucial site in the N-terminus (K85) and several checkpoints in the MTR are predominantly involved in the establishment of an opening-permissive pore geometry and to a lesser extent or not at all in STIM1 coupling (Table 3). Indeed, the K85E mutation allowed still activation under certain conditions, for instance, when attached to two STIM1-CAD fragments (-SS) (e.g., Orai1 K85E-SS or Orai1 K85E H134A-SS) [76,82] or within an Orai1 dimer: Orai1 K85E–Orai1 H134A [82]. Contrarily, all kinds of E149K or L174D mutants led to LoF [82] (Table 3). Thus, in addition to the Orai1 C-terminus being the main coupling site for STIM1, the loop2 region is clearly involved in the interaction with this ER-located Ca^2+^ sensor. The cytosolic loop region likely acts as a bridge for the communication with the Orai1 N-terminus and, thus, the pore [82].

Taken together, Orai pore opening requires collective TM domain motions which seem to be conveyed by the interaction of STIM1 with the Orai1 C-termini and loop2. Whether these global structural alterations are triggered via a direct or allosteric communication of STIM1 with the Orai1 N-terminus remains to be determined. 

### 3.4. Pathophysiological Roles of Orai1 GoF and LoF Mutants

The correct function of Orai1 is indispensable for the maintenance of healthy processes. Gene mutations in these CRAC channel components can cause diseases resulting in severe pathologies. In the last decade, the identification and examination of a multitude of disease-related single-point mutations have been of the utmost importance. Gained insights can be used to design novel drugs for treating these diseases. 

Among the series of known Orai1 GoF and LoF mutations within the four TM domains some are related to diverse diseases (Figure 3; Table 1). A screen through a cancer genome database together with functional studies revealed several constitutively active Orai1 single-point mutants (Orai1 H134A, Orai1 A137V, Orai1 L138F, and Orai1 M139V) in TM2 that appear in various human cancers, such as colorectal adenocarcinoma or stomach carcinoma [44]. Bulla et al. [99] discovered three GoF mutations within TM1 and TM3 of Orai1 (Orai1 G98S, Orai1 V107M, Orai1 T184M) that are connected to the tubular aggregate myopathy (TAM). The constitutively active mutants cause pathologies such as muscle weakness, contractures, and miosis. Interestingly, in contrast to Orai1 T184M in TM3, only the mutants Orai1 G98S and Orai1 V107M, located in the channel pore, are constitutively open without the presence of STIM1. The Orai1 mutation P245L, located in TM4, is also associated with Stormorken-like syndrome [5], indicating the inevitability of the proline at this position for Orai1 gating. A second GoF mutation in TM4, Orai1 G247S, leads to neck carcinoma [44]. Interestingly, a loop2 GoF mutation (Orai1 S159L) is also related to uterine carcinoma [44]. Orai1 R91W, Orai1 G98R, and Orai1 A103E, all located in TM1, are LoF mutations that cause immunodeficiencies [7,96,98]. LoF mutations in TM3, Orai1 V181SfsX8 and L194P, lead to autoimmunity and ectodermal dysplasia [96,98], while Orai 1 G183D causes glioblastoma [44] (Figure 3 and Table 1).

The distribution of disease-inducing GoF and LoF mutations over the whole Orai1 protein is in support of the assumption that Orai1 activation is associated with a global conformational change of the Orai1 complex. Altogether, the precise interplay of Orai1 and STIM1 is necessary to ensure normal physiology in the human body. Only for some of these disease-causing mutations is the mechanism of how they exactly disturb the channel function known [44,92,102].

## 4. Essential Orai Gating Checkpoints

Essential Orai1 gating loci that control the open and closed conformation of the channel are located in the transmembrane regions. As already indicated above, due to their location either in the middle TM segment or at the interface of the cytosol and the membrane, we recently assigned them to either the MTR or the CETR (Figure 4). The MTR contains as crucial gating sites a hydrophobic cluster at the TM1–TM2/3 interface and a set of checkpoints controlling the maintenance of the closed state and an opening-permissive Orai1 conformation in the conical MTR ring (including the H134 residue and several others) (Figure 4A). The CETR includes as essential gating checkpoints the nexus region connecting TM4 and the C-terminus, the hinge plate formed between TM3 and TM4, and the cytosolic triangles established between loop2 and the N-terminus (Figure 4B). Another essential gating region formed at the TM1–TM2/3 interface represents a serine ridge that spans from the MTR to the CETR (Figure 4C). Moreover, the TM2/TM3 ring forms a rigid body between TM1 and TM4 across the membrane (Figure 4C) [28,39,54,78,82,88,97]. The individual regions and their roles in the STIM1/Orai1 choreography are described in detail in the subsequent sections.

### 4.1. Gating Checkpoints in the MTR

The MTR includes more than a dozen residues (Figure 4A), which, when mutated, can become constitutively active. They all have in common that they control the closed and open state of the Orai channel, and they are not required for functional STIM1 coupling (Table 3). Not all but some of those further maintain signal propagation to the pore and/or an opening-permissive pore geometry. In addition, a hydrophobic cluster including a functional relevant sulfur–aromatic interaction controls an opening permissive pore conformation [39,82,97].

#### 4.1.1. MTR of TM2/TM3 

The best-described gating hotspot, H134 (Figure 4A), is located within the middle TM2 region and faces the non-pore-lining residues of TM1 [36,39,44]. It predominantly controls an opening-permissive conformation of the pore, as well as the channel complex [39,44,82]. Orai1 H134A/C/S/T exhibit constitutive activity with biophysical properties almost comparable to CRAC channel currents [39,44]. Only FCDI is not maintained, as shown for Orai1 H134S [39]. Mechanistically, Frischauf et al. [44] proposed that H134 might support the closed state by forming hydrogen-bond interactions with TM1, specifically S93 and S97. Consequently, they assumed that the disruption of those interactions initiates gating by switching the channel conformation to the open state [44]. In accord, the recently published high-resolution cryo-EM structure of dOrai H206A suggests that this histidine substitution eliminates the hydrogen bond with S93 and abolishes van der Waals interactions [51]. The study by Yeung et al. [39] reported that H134 operates as a steric brake. They showed, for a set of Orai1 H134X (X = any amino acid) mutants via a relation of the current density and side-chain surface area, that rather a smaller side-chain size of the substituted residue induces spontaneous channel activation. Moreover, they identified no correlation of constitutive channel activation and hydrophobicity of the respective inserted residue. Yeung et al. [39] described that substitution of H134 to a serine or threonine which have hydroxyl groups predictably available for hydrogen-bond formation induces robust constitutive activation of the channel. Thus, the hydrogen-bond interaction between H134 in TM2 and S93 and S97 in TM1 does not necessarily account for the closed state of Orai1. Instead, the bulky histidine seems to represent a steric brake which is released at the TM1–TM2 interface upon its substitution to a smaller residue, subsequently allowing the channel to open. Yeung et al. [39] further proposed that H134 interacts via hydrogen bonds with the TM2 residue L130 located one helical turn above. The latter interaction was hypothesized to stabilize the H134 side chain to keep facing toward the TM1–TM2/3 ring interface in the Orai1 quiescent state. 

Constitutive activity of Orai1 H134S was shown to be accompanied by a rotation of TM1 within the hydrophobic cavity. Cd^2+^ permeation experiments using Orai1 G98C H134S and Orai1 F99C H134S revealed that F99C is moved away from the pore axis in the Orai1 H134S mutant independent of the presence of STIM1 [39]. Moreover, H134S was introduced in the Orai1 V102C mutant to form an Orai1 V102C H134S double mutant. Remarkably, H134S enabled regaining the Ca^2+^ selectivity of Orai1 V102C comparable to Orai1 V102C in the presence of STIM1 [39]. In accordance with these findings, MD simulations also revealed a counterclockwise pore helix rotation [44,82,88]. Whether other CRAC channel hallmarks are also retained has so far not been investigated. Moreover, other constitutively active Orai1 mutants, containing the substitution in TM3 or TM4, could be tested for an effect on the reorientation of G98 and F99.

Among the different amino acids screened for their effects when inserted at position H134 in Orai1, not only GoF mutants were identified, but also some LoF mutants (e.g., H134W). This suggests that this position is not only involved in the maintenance of the closed state of Orai1, but also in establishing an opening-permissive pore and channel conformation [39,44,82].

In addition to Orai1 H134A, a series of other GoF mutations were identified in the MTR [39,44,82,102] (Figure 4A), suggesting that they are also involved in the maintenance of the closed state. Only at some positions, substitutions to amino acids with distinct properties lead to LoF highlighting their additional role in the establishment of an opening-permissive conformation (e.g., H134, V181). Interestingly, single-point mutations of L185 similar to P245 either lead to GoF or maintain store-operated activity, but do not lead to LoF. It is tempting to speculate that the diverse gating hotspots in TM2 and TM3 act similarly to H134 as steric brakes, presumably keeping the channel in the closed state. These checkpoints potentially interplay in an allosteric manner with each other, as demonstrated for instance by Yeung et al. [39] for Orai1 L130. However, how they interfere with pore opening still needs to be determined.

Interestingly, our recent study [82] revealed that double-point mutants containing a combination of one LoF and one GoF mutation in the MTR, independent of their location relative to each other, always lead to LoF (Table 2). In line with these findings, double mutants exhibited similar crosslinking of pore-lining residues such as wildtype Orai1. Furthermore, in MD simulations, rotation of the hydrophobic region in the pore-lining TM1 domain was also only observed for Orai1 H134A, but not for the corresponding double mutant additionally containing a LoF mutation within one of the TM domains [82]. This highlights that Orai1 pore opening necessitates global conformational changes of the entire channel complex [82] (Table 2 and Table 3).

Summarizing, a series of gating checkpoints in the MTR control an opening-permissive pore conformation and require clearance to guarantee pore opening. 

#### 4.1.2. The Hydrophobic Cluster at the TM1–TM2/3 Ring Interface 

The interface of TM1 and the TM2/TM3 ring situated directly across the pore-facing F99/V102 hydrophobic region and at the level of the selectivity filter (E106) is lined by the tightly packed hydrophobic cluster including residues L96, M101, M104, V105, F123, F187, V191, and L194 (Figure 4A). The latter interface has been determined by the atomic packing analysis as the locus with the highest packing density [39]. This cluster has been suggested to control the opening and closing of the Orai1 channel gate. In support, the substitution of these residues to small or polar amino acids abolished CRAC channel gating without impairing STIM1 binding [30]. This indicates that disturbances within the hydrophobic cluster hamper the gating signal from effective transmission from the TM2/3 ring to the pore. Thus, the hydrophobic stack seems to represent a crucial component of the STIM1-mediated conformational motions which induce activation of Orai1 channels. Consequently, the disrupted hydrophobic interactions alter the communication between the TM1 pore region and the surrounding helices. Specifically, a sulfur–aromatic interaction between M101 and F99 of adjacent subunits was reported to trigger channel activation. In contrast, in the closed state Orai1 is supposed to form interactions of M101 and F187 [97]. To what extent other residues in the hydrophobic cluster affect pore opening remains to be determined. 

#### 4.1.3. Kink in TM4 and Outer TM4 Segment

A prominent gating checkpoint located at the kink of TM4 represents P245. Its single-point mutation to any other amino acid always results in a constitutively active channel, which highlights the role of P245 in the maintenance of the quiescent state of the Orai channel [102]. Mutagenesis screens within Orai1 TM domains [39,82] revealed, in addition to P245, A235, S239 and F250 (Figure 4A) as the most prominent positions involved in the maintenance of the closed state. Interestingly, a cysteine substitution of A235 or S239 leads to GoF, while a tryptophan mutation causes LoF [39,82]. Altogether, these positions are required for controlling the open and closed pore and ensure signal propagation and/or pore hydration, while they do not interfere with STIM1 coupling [82]. However, additional investigations are still required to clarify how these gating checkpoints contribute to the maintenance of the closed state of Orai1. Do they function as steric brakes similar to H134 [39], or do they form interactions with TM3? Noteworthy, residue A235 is juxtaposed with C195 and F199 in TM3, whereas S239 is positioned close to the TM3 amino acids V192, C195, and W196. These residues seem to form critical interactions between TM4 and TM3 within Orai1. Moreover, investigations are still required to determine whether cysteine crosslinking between C195 and one of the two point mutations, A235C or S239C, potentially induces constitutive activity. 

Similar to the LoF mutations in TM2 and TM3, the MTR LoF mutants Orai1 A235W and Orai1 S239W act dominant over a series of GoF mutants (Table 2). Moreover, constitutive activity of diverse GoF mutations in TM4 (e.g., A235C, S239C) is abolished by a series of LoF mutations, both in the MTR and in the CETR. This highlights again that pore opening necessitates clearance of a series of gating checkpoints and concerted TM domain motions [82]. 

### 4.2. Gating Checkpoints in the CETR

Several known gating checkpoints in the CETR (Figure 4B) have in common that they control STIM1 coupling and signal propagation or even an opening-permissive pore conformation (Table 3). One checkpoint (hinge region) at the channel periphery is additionally involved in the maintenance of the closed state of the Orai1 channel [82].

#### 4.2.1. The Major Role of the Orai1 Hinge Region in CRAC Channel Activation

A prominent gating hotspot represents the sharp bent region connecting the Orai1 C-termini and the TM4 which is known as the gating nexus (aa 261–265) (Figure 4B). Specifically, the nexus segment consists of the hinge plate (LV, aa 261–262) and the bent domain (SHK, aa 263–265) [28,78]. This bent region is assumed to possess two roles: (1) establishment of functional STIM1 binding and (2) control of the closed and open state of Orai1 channels. In support, LoF (e.g., L261D, _262_AAA_264_, _262_GGG_264_) and GoF (e.g., L261A, V262N) mutations interfere with STIM1 coupling [28,79,83]. Moreover, the nexus GoF mutants, including a two- (L261A, V262N) and fourfold mutation (L261A, V262N, H264G, K265A; Orai1 ANSGA), induce constitutive Orai1 channel activity [78]. Their constitutively active currents display biophysical characteristics (see Section 2.1) highly comparable to that of the STIM1-activated wildtype Orai1 channel [78]. Nevertheless, it is worth mentioning that the extent of FCDI, as well as of reactivation, typical for STIM1-mediated Orai1 currents is less pronounced for constitutive Orai1 ANSGA currents [32,39,54]. Despite these differences in biophysical characteristics of Orai1 ANSGA and STIM1–Orai1-mediated currents, it is proposed that Orai1 ANSGA similar to Orai1 H134A captures an open state matching best that of the STIM1-bound Orai1. Altogether, these data indicate that alterations within the hinge region affect both the channel’s active state and STIM1 coupling. 

Functional changes due to mutations within the nexus might be explained by structural reorientations within this region [105]. Indeed, a comparison of the closed and the open crystal structures of dOrai [34,35,36,51] suggests that the kinked, antiparallel oriented C-termini in the quiescent state straighten toward the cytosol via unlatching conformational changes. Nevertheless, whether such structural alterations are occurring physiologically and which conformational changes occur actually in Orai1 nexus mutants still need to be investigated in more detail.

A combination of the ANSGA mutation in Orai1 with diverse LoF mutations located both in the CETR and in the MTR completely abolished the constitutive activity of this Orai mutant channel. Thus, a series of LoF mutations act dominant over GoF mutations within the nexus (Table 2 and Table 3). Interestingly, vice versa, certain LoF mutations in the nexus possess no dominance over constitutively active point mutations in the MTR (e.g., H134A). Hence, while the maintenance of constitutive activity of Orai1 ANSGA requires a series of sites in the MTR and CETR to capture an opening-permissive conformation, other constitutively active mutants (e.g., Orai1 H134A) retain function independent of an intact nexus [82] (Table 2 and Table 3). These findings suggest distinct activation pathways for the Orai1 ANSGA compared to a variety of MTR GoF Orai1 mutants (e.g., Orai1 H134A). Whether the signal propagation pathway of STIM1-mediated Orai1 activation is rather comparable to Orai1 ANSGA or Orai1 H134A remains to be determined. 

In summary, the nexus LVSHK (L261, V262, A263, H264, K265) is essential in STIM1-mediated Orai1 activation, specifically for STIM1 coupling, maintaining the closed state and controlling an opening-permissive conformation. It might be assumed that local conformational changes within the nexus trigger global conformational changes to finally establish pore opening.

#### 4.2.2. Hinge Plate—The Hydrophobic Interface of TM3–TM4 Critical in Gating 

The role of the hinge region in pore opening is further supported by the interplay of hydrophobic residues in TM3 and TM4 (Figure 4B). A key communication link between TM3 and TM4 is established by the residues L261 in TM4 and L174 and A175 in TM3 located opposite to each other. The interplay of these hydrophobic residues controls (1) STIM1 binding and (2) pore hydration [82]. Indeed, their mutation to basic or acidic residues significantly reduced (Orai1 L261D/K) or abolished (Orai1 L174D/K; A175D/K) STIM1-induced Orai1 activation [78]. MD simulations revealed a dewetting of the hydrophobic part of the pore [82]. STIM1 coupling to those mutants was partially reduced for Orai1 L174D/K [78]. This indicates that disturbances of TM3–TM4 interactions can alter the STIM1 binding site in line with the findings on Orai1 ANSGA. Accordingly, STIM1 was recently reported to gate Orai1 channels not only via coupling of their C-termini, but also via communication with an Orai1 loop2 region in close proximity to L174 [81]. 

Moreover, we recently discovered a dominant effect of the LoF mutation L174D over a series of GoF Orai1 mutations independent of the presence of STIM1. Indeed, L174D impacts pore hydration not only of wildtype Orai1, but also of the GoF Orai1 H134A, in addition to its interference with STIM1 coupling. This highlights the dual role of L174 in adjusting an opening-permissive pore conformation and controlling STIM1 coupling [82] (Table 2 and Table 3). Moreover, these investigations are proof that Orai1 pore opening necessitates global conformational changes within the channel complex. 

Accordingly, disulfide-mediated crosslinking between Orai1 L174C and Orai1 L261C increased STIM1-induced channel gating, underlining the importance of the TM3–TM4 interactions for Orai1 gating. In support, Liu et al. [34] reported that TM3–TM4 hydrophobic interactions are essential for STIM1-mediated Orai1 activation. Specifically, they showed that F178 (F178A) in TM3 and F257 (F257A) and L261 (L261D) in TM4 ensure STIM1-mediated Orai1 activation, but not STIM1 coupling [34]. 

Several other hydrophobic residues located more in the center of TM3 and TM4, W176, V181, L185, and F187 within TM3 and F250 within TM4, have been reported to contribute to the maintenance of the closed state of the channel [39,43,54,82] (Figure 4A,B). Their single-point mutation to a small amino acid such as alanine, serine, or cysteine led to constitutive activity. Their authentic CRAC channel hallmarks are only fully preserved in the presence of STIM1 [54]. Only Orai1 W176A/C exhibited constitutive nonselective currents, which did not regain high Ca^2+^ selectivity upon the co-expression of STIM1 [100]. Remarkably, while Orai1 V181A or Orai1 L185A displayed small, constitutively active Orai1 currents, a double-point mutation including L185A in TM3 and the oppositely located F250A in TM4 showed strongly elevated constitutive activity [54]. 

Interestingly, the quiescent dOrai structure reveals a close positioning of TM3 and TM4 [35], whereas, in the open structures (dOrai H206A, dOrai P288L), these helical regions seem to be located farther apart [34,36]. These structural differences indicate, on the one hand, that the closed Orai1 state is maintained as long as TM3 interacts with TM4b. On the other hand, pore opening probably involves a conformational change along TM3 and TM4b [36]. Interestingly, computational approaches by Dong et al. using a putative open-state structure of dOrai [41] revealed similar rearrangements of TM4 helices as so far described for the crystal structures; nevertheless, the tight packing between L174 within TM3 and L261 within TM4 remained almost fully preserved. This observation is in line with the previously mentioned cysteine crosslinking experiments (L174C–L261C) performed by Zhou et al. [78]. This would indicate that the hydrophobic packing between TM3 and TM4 is required for an opening permissive conformation contrary to the assumptions made from the dOrai crystal structures.

Concluding, the communication between TM3 and TM4 is essential for STIM1 coupling, the transmission of the STIM1-mediated activation signal at the periphery of the Orai1 complex toward the pore, and pore opening. In addition to the reported L174–L261 [78] interplay upon STIM1-induced conformational changes in the nexus, it is probable that further TM3–TM4 interactions are formed or released upon Orai1 activation. This might involve, for instance, V181, L185 (TM3) and F250, F253 (TM4). However, it is still elusive how these positions or even additional ones in TM3/TM4 contribute to the maintenance of the Orai1 closed state. Moreover, it remains to be determined how a pore-opening-permissive conformational change takes place along TM3 and TM4. 

#### 4.2.3. Communication between N-Terminus and Loop2 Established by Cytosolic Triangles 

The hydrophobic interface formed by TM3 and TM4 is directly connected via the cytosolic loop2 region to the Orai1 N-terminus (Figure 4B). Thus, it is likely that Orai1 activation involves the signal transmission from Orai1 C-terminus via the hinge plate and the loop2 region finally to the pore. Indeed, as outlined below, both the cytosolic loop2 region and the N-terminus are crucial for both STIM1 coupling and Orai1 pore opening. 

We discovered that the maintenance of STIM1-mediated Orai1 activation requires an opening-permissive communication of the N-terminus and loop2. Artificially induced close proximity of the N-terminus and loop2 in full-length Orai1 via cysteine crosslinking (K78C–E166C) significantly reduced store-operated currents. Moreover, LoF of certain Orai1 N-truncation mutants underlies an inhibitory interaction of the N-terminus and the loop2 region, as shown via MD simulation and atomic force microscopy. Release of these inhibitory interactions partially recovered Orai1 function [43]. 

In extension, STIM1-mediated gating involves the formation of intra- and inter-Orai subunit salt-bridge interactions between acidic and basic residues in the ETON region and the cytosolic extended portions of TM2 and TM3. Specifically, K85 with E173 and R83 with E149 form salt-bridge interactions within one subunit [41,82], while K85 with E149 forms an inter-subunit salt bridge [82]. Indeed, we showed, in line with Dong et al. [41,82], that, while Orai1 K85E and Orai1 E149K lead to LoF, charge reversal double-point mutants Orai1 K85E E173K and Orai1 R83E E149R/K exhibited STIM1-mediated activation and at least partially recovered STIM1 coupling. Concerning the third inter-subunit salt bridge K85–E149, a charge reversal mutant Orai1 K85E E149K does not regain function, but its role becomes clear via comparison of the effects of a series of point mutants of basic and acidic residues. Interestingly, the single-point mutants Orai1 R83E and E173K displayed preserved, although slightly reduced, STIM1-mediated activity, despite one potentially expecting similarly abolished salt-bridge interactions as for the two LoF mutants Orai1 K85E and Orai1 E149K. This represents that the interplay of K85 and E149 of adjacent subunits is also crucial for the maintenance of STIM1-mediated Orai1 channel activation, as we distilled via extensive site-directed mutagenesis completed via MD simulations [82]. At least two of the three salt-bridge interactions need to be intact to allow STIM1-mediated Orai1 gating. The evidence for the functional relevance of the salt-bridge pair R83–E149 is still puzzling as they are located in both closed and open dOrai structures, 14 Å apart from each other. Moreover, R83 points into the pore and, thus, away from E149 [82]. In accordance, MD simulations revealed that Orai1 gating motions involve clockwise reorientation of R83 toward E149 [41]. STIM1 coupling likely promotes the formation of this salt bridge, thus stabilizing the open state. Indeed, we discovered that introduction of the charge reversal mutation R83E E149K in Orai1 H134A only regained activity in the presence, but not in the absence of STIM1. This highlights the role of STIM1 in promoting this salt-bridge interaction [82]. However, further proof for a potential rotation of TM1 induced via STIM1 is still required.

Similar to L174 in the hinge plate [82], as well as residues in the basic region of the pore [37], K85 and E149 are also indispensable for sufficient pore hydration, both in wildtype Orai1 and in Orai1 GoF mutants. The dominant effect of the LoF mutations K85E and E149K over diverse GoF mutations in the MTR region emphasizes that concerted TM domain motions ensure pore opening [82] (Table 2 and Table 3). 

Additionally, a recent study on *Caenorhabditis elegans* CRAC channels determined a distinct gating mechanism compared to that in mammals. Noteworthy, cSTIM or cCAD coupling to the loop2 of cOrai was confirmed to be sufficient to gate the channel [106]. This suggests that the gating process of SOCE has evolved between different organisms over time.

In addition to the interplay of Orai1 N-terminus and loop2, the N-terminus itself, especially the conserved, helical extension of TM1 (ETON region, aa 73–90) is crucial for STIM1-mediated gating, as shown via a series of N-truncation mutants. Moreover, it controls authentic CRAC channel hallmarks, which are summarized in Section 2.1 [54,64,74]. 

Summarizing, the N-terminus and loop2, as well as their opening-permissive interplay, govern STIM1 coupling and pore opening, as well as fine-tune biophysical features of CRAC channel currents. The communication of the N-terminus and loop2 is established via a set of electrostatic interactions [41,82].

### 4.3. Gating Regions Spanning from the MTR to the CETR

#### 4.3.1. TM2/3 Ring Boost Cooperativity in STIM1-Mediated Orai1 Activation

As described above, TM2 and TM3 are sandwiched between the TM1 and TM4 domains within the Orai channel complex [35] (Figure 4C). They are supposed to form a single unit of tightly interlocked TM2 and TM3 regions essential in transmitting the Orai1 activation signal from the peripheral STIM1 coupling site to the gate of the channel. While the communication of the TM2/TM3 with TM1 is already well understood, its interplay with TM4 is still elusive. Both TM2 and TM3 contain several crucial gating checkpoints, with H134 as the most outstanding position communicating with TM1. Moreover, the hydrophobic cluster and a serine ridge (see next paragraph) represent essential links between TM1 and TM2/TM3 [39]. A more detailed investigation of three residues in or close to the hydrophobic cluster suggested that sulfur–aromatic interactions determine the closed or open state of Orai1 channels. While, in the closed state, the interaction of M101 and F187 persists, in the open state, F99 forms a sulfur–aromatic interaction with M101 of the adjacent subunit [97]. Referring to an interplay of TM2/TM3 with TM4, mutation of residues in the TM2/TM3 ring and TM4 can lead to GoF (e.g., S239C) or LoF (e.g., S239W). These and further communication sites still require more detailed investigations. A key question is still whether pore opening is associated with an enhanced or decreased distance between TM3 and TM4.

Moreover, this TM2/TM3 ring together with the sulfur–aromatic interaction of TM1/TM2 was suggested to enforce cooperativity in the STIM1-mediated channel activation process [39,97]. On the one hand, unbinding of the TM2 and TM3 helices was predicted to be energetically highly unfavorable. On the other hand, concatemeric studies revealed that a single defect of the STIM1 coupling site within the Orai complex strongly reduces the probability for CRAC channel activation [87]. These findings led to the assumption that STIM1 coupling to each of the Orai1 C-termini leads to signal transmission via the TM2/3 ring to all six pore helices. Thus, similar to the MTR and CETR, the tightly interlocked TM2/3 helices can also be described via the working mechanism of the logical element “AND” gate. 

#### 4.3.2. Serine Ridge at the Intersection of TM1 and the TM2/TM3 Ring

A ridge of small, polar serine chains (S82, S89, S90, S93, S97) is located along TM1 from the MTR to the CETR (Figure 4C). Yeung et al. [39] proposed that this serine ridge could provide sufficient conformational flexibility to the Orai1 pore required for intact channel gating. Atomic packing analysis revealed that the serine ridge is established by polar serines and alternating stripes of hydrophobic residues [39]. This observation correlates with the pattern of polar–hydrophobic stripes at the TM2/TM3 ring, indicating that mutations that enhance the hydrophobicity of the serine ridge might induce interactions with a hydrophobic segment within the TM2/TM3 ring. The human GoF mutation Orai1 S97C associated with the Stormorken-like syndrome was proposed to lead to constitutive activity due to disrupted polar interactions between TM1 and the TM2/TM3 ring responsible for stabilizing the closed state [39]. Moreover, the single substitution of S97 by hydrophobic or large residues (S97C/M/L/I/V) revealed nonselective currents in the presence of STIM1 [39]. Thus, it can be assumed that the hydrophobic point mutation disorganizes the alternating polar/nonpolar interface with the TM2/TM3 ring. This limits the flexibility of the pore helix capturing Orai1 in a nonselective open state. A similar scenario could potentially also explain the behavior of two disease-related mutants Orai1 A137V (colorectal carcinoma; [44]) and L138F (myopathy; [95]) which both exhibit constitutive partially nonselective currents. As A137 faces toward S93 and L138 faces toward S93 and S90 in Orai1, it can be assumed that the polar interactions between TM1 and the TM2/TM3 ring stabilizing the closed state are disrupted in the respective constitutively active point mutations. However, experimental proof is still required. Furthermore, the role of other serines located in the ETON region, especially S82 located opposite to L145 still requires further investigations. 

## 5. Isoform-Specific Orai Gating

The three Orai isoforms, Orai1, Orai2, and Orai3, are all activated in a store-operated manner by STIM1 coupling and display high Ca^2+^ selectivity with a reversal potential of +50 mV [58]. The subunits of each isoform are all composed of four TM domains encompassed by the N- and C-termini and connected via one intra- and two extracellular loops [107,108,109]. Nevertheless, they feature several distinct functional and structural properties, as outlined below. 

While TM1 (aa 92–106) is fully conserved among all Orai isoforms, TM2 (aa 118–140), TM3 (aa 174–197), and TM4 (aa 236–258) share ~81–87% sequence identity. Major isoform-specific structural differences occur within the cytosolic and extracellular regions [52]. Specifically, the cytosolic N- (aa 1–90) and C-terminal (aa 265–301) strands show only 34% and 46% sequence identity, respectively [110]. In particular, the cytosolic extended region of TM2 has been suggested to be shorter in Orai1 than in Orai3, thus leading to a longer flexible loop2 segment between TM2 and TM3 in Orai1 [43].

At the functional level, one difference is reflected in STIM1-mediated maximum Orai current densities, which are 2–3-fold higher for Orai1 than for Orai2 and Orai3 [26,58]. These distinct current levels likely underlie polybasic- and proline-rich domains only present in the Orai1 N-terminus [111]. Their mutation [21,73,112] significantly lowered Orai1 Ca^2+^ currents to levels comparable to those of Orai2 and Orai3.

STIM1-mediated Orai currents all exhibit FCDI, although with isoform-specific variations. Orai3 channel currents exhibit three times more pronounced FCDI than those of Orai2 and Orai1 [58,66,113]. Additionally, upon application of a hyperpolarizing voltage step over 2 s [58,66], STIM1-mediated Orai1 currents typically display subsequent to a fast inactivation phase within the first 100 ms, with a reactivation phase reaching its maximum after ~1500 ms. In contrast, the fast inactivation phase of STIM1-mediated Orai2 and Orai3 currents is followed by a slow inactivation phase [58,66,113]. These distinct extents in FCDI can be explained by diverse structural alterations within the cytosolic strands and the loop2 region [113,114]. Interestingly, inactivation of native CRAC currents is more pronounced than that of Orai1 currents and reactivation is lacking, which can at least be partially explained by the potential formation of heteromeric Orai channels [24,65]. 

Moreover, the well-known enhancements of currents in a DVF versus a Ca^2+^-containing solution are in principle preserved for all Orai isoforms. Nevertheless, the ratio of I_DVF_:I_Ca^2+^_ is higher for STIM1-mediated Orai3 compared to Orai1 currents. The reason for that is likely the more pronounced FCDI of Orai3 versus Orai1 in a Ca^2+^-containing solution [53]. 

In addition, the sensitivity to the chemical compound 2-aminoethyldiphenyl borate (2-APB) occurs in an isoform-specific manner [58,115,116]. Native CRAC, STIM1-mediated Orai1 and Orai2 currents are abolished by 50 µM 2-APB. Contrarily, the application of 2-APB to Orai3 currents leads to huge double rectifying currents independent of the absence or presence of STIM1 [117]. Among two other drugs, Synta66 was discovered to block Orai1, but increase Orai2 currents, while IA64 boosts Orai1 and decreases Orai2 currents [118].

Structural properties that determine Ca^2+^ permeation and Ca^2+^ selectivity in the CAR region are also distinct within the Orai homologs. Orai1 contains three glutamines, whereas Orai2 and Orai3 possess a mixture of glutamines, glutamates, and aspartates. While homomeric Orai channels remain undisturbed by these differences, heteromeric Orai1/Orai3 complexes exhibit diminished Ca^2+^ selectivity and enhanced Cs^+^ permeation [66,119].

Furthermore, supercoil regions within the C-termini of Orai channels also contribute in an isoform-specific manner to their activation. Specifically, STIM1 coupling is suggested to occur with distinct affinities to the helical coiled-coil regions in the Orai C-termini. This assumption is based on the effects of site-directed mutagenesis. A single-point mutation in the Orai1 C-terminus (L273S/D) or in the counterpart of the STIM1 C-terminus (L373S) is sufficient to abolish their coupling to and activation of Ca^2+^ entry via Orai1. Orai2 and Orai3 require a double-point mutation in their C-termini or within the STIM1 C-terminus (L373S A376S) to fully abolish their interplay [26,72,73].

Moreover, the communication of the Orai1 N-terminus and the loop2 region depends on isoform-specific differences [64]. This becomes clearly evident from Orai1 and Orai3 N-truncation mutants. Despite the ETON region being fully conserved, Orai1 requires a five residue longer portion of this region compared to Orai3 to maintain STIM1-induced activation [64]. The reason for that underlies distinct properties of the Orai loop2 regions. Our chimeric approach revealed that STIM1 coupling and the function of the inactive Orai1 N-truncation mutants such as Orai1 ΔN_1–78_ was restored upon swapping the loop2 region between TM2 and TM3 with that of Orai3 (Orai1 ΔN_1–78_ Orai3 loop2) [43]. In analogy, the exchange of only five nonconserved residues within Orai1 loop2 with those of Orai3 loop2, restored STIM1-mediated activation of the N-truncation mutants. Vice versa, an analogue Orai3 N-truncation mutant (e.g., Orai3 ΔN_1–53_) lost store-operated function upon the swap of Orai1 loop2. Isoform-specific features of the loop2 regions underlie distinct structural properties as shown via MD simulations. While Orai3 possesses a longer TM2 cytosolic helical extension compared to Orai1, the flexible region between TM2 and TM3 is shorter in Orai3 compared to that in Orai1. Thus, the overall structure and flexibility of the region connecting TM2 and TM3 regulate Orai N-truncation mutant functionality. This indicates that the intracellular loop2 of the two Orai isoforms possesses distinct affinities to STIM1. 

Orai1 constitutive mutants that lose activity due to N-truncations also regain function upon the swap of Orai3 loop2 independent of STIM1 [43]. Interestingly, this holds not for Orai1 ANSGA, as lost function upon its N-truncation cannot be restored by the swap of Orai3 loop2. This indicates distinct requirements in gating loci for Orai1 compared to Orai3 [32].

Among a diversity of currently known constitutively active mutants, we discovered a dramatic difference in the extent of currents for the two analogue mutants Orai1 L185A and Orai3 F160A. While constitutive currents of Orai1 L185A are much smaller than those of STIM1-mediated Orai1 currents, Orai3 F160A exhibits huge constitutive activity in comparison to STIM1-mediated Orai3 currents [54]. Interestingly, two residues in TM3 are not conserved in Orai1 and Orai3: V181 and L185 in Orai1 and A156 and F160 in Orai3. Whether these two positions are solely responsible for the observations still requires continuing investigations. It is likely that signal propagation within the Orai isoforms follows distinct pathways [43,54]. These critical residues of the two Orai isoforms in TM3 are located opposite to TM4. At this point, it still requires further examination if mutations in TM3 leading to constitutive activity induce conformational changes such as the movement of TM3 closer to or farther away from the adjacent TM4. 

Lastly, oxidative stress controls Orai channel function via TM3 in an isoform-specific manner. The reason for that represents a nonconserved residue in TM3 (C195 in Orai1) and the analogue G170 in Orai3. Orai1 but not Orai3 currents are inhibited by H_2_O_2_ [120]. This inhibitory action underlies altered subunit interaction within the oxidized Orai1 channel [121].

In summary, while general activation mechanisms of the three Orai isoforms are similar, they vary in functional, structural, and pharmacological properties. These differences might provide targets for isoform-specific interference with Orai channel function, potentially required in the treatment of Orai isoform-related diseases. 

## 6. Summary, Open Questions, and Perspectives

**Figure 6:**
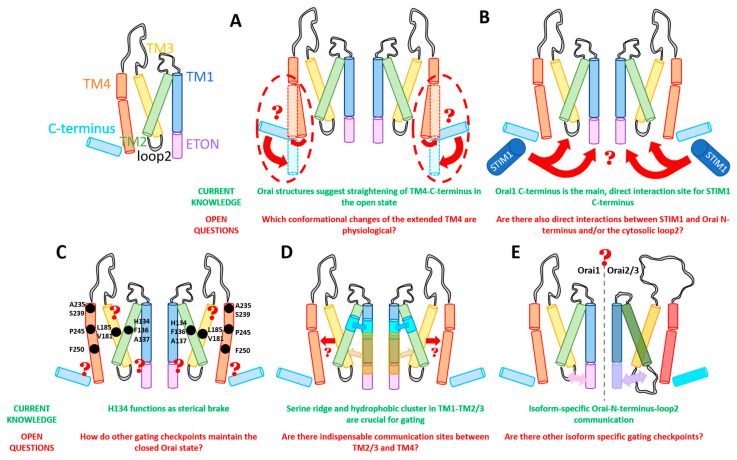
Open questions. The simplified model scheme summarizes the current knowledge of Orai1 channel gating and so far open corresponding questions. Scheme of one Orai subunit with the the main regions indicated. (**A**) The available dOrai structures suggest a straightening of the TM4-C-terminus regions, however, whether those changes are also occurring physiologically needs to be confirmed. (**B**) The direct interaction of STIM1 with the C-termini of Orai was proven. Moreover, the loop2 region communicates with STIM1 in relaying a gating signal to the pore. While the interaction site of STIM1 and Orai1 C-terminus is well-defined, potential binding pockets of STIM1 with the Orai1 N-terminus or the loop2 still need to be resolved. (**C**) Position H134 is the best characterized gating checkpoint within Orai1 likely functioning as a steric brake to maintain the closed state of Orai1. How this principle is applicable to other gating checkpoints is still unknown. (**D**) While the interacting regions between TM1 and TM2/3 are well-defined, not so much is known about the interface formed by TM2/3 and TM4. (**E**) Several isoform-specific features of Orai channels have been identified. Whether Orai channel activity is controlled via additional sites in an isoform-specific manner remains to be investigated.

Since the discovery of the molecular components of the CRAC channel, substantial progress in the understanding of a series of activation steps of the unique STIM/Orai activation choreography has been achieved [32,33]. Nevertheless, diverse aspects within the activation cascade of the STIM and Orai proteins are still elusive (Figure 6). 

Highly valuable structural resolutions of dOrai consistently reveal that pore opening involves dilation of the basic region [34,35,36,51]. However, which conformational changes at the Orai complex periphery occur physiologically still need to be identified. Meanwhile, two long-awaited cryo-EM dOrai structures [34,51] are available in addition to several dOrai crystal structures. However, for a better understanding of conformational changes occurring upon the transition from the closed to the open state, structures with higher resolution in the presence of STIM1 are still overdue (Figure 6A). 

Additionally, it is known that Orai C-termini represent the main coupling site for STIM1 C-termini. The Orai loop2 regions are crucial gating sites addressed by STIM1. However, whether the Orai1 N-terminus also functions as a direct interaction site with STIM1 is still unknown. Moreover, a detailed mapping of STIM1 C-terminus–loop2 and STIM1 C-terminus–N-terminus binding pockets is outstanding (Figure 6B). 

An arsenal of gating checkpoints is currently known, and all require clearance to allow pore opening. Their impact on each other, as we revealed recently via a systematic screen on double-point mutations [82], is proof that a global conformational change within the entire Orai channel complex is indispensable for pore opening. However, among the series of checkpoints, only a few (e.g., the role of H134) are currently understood in detail [37,39,97]. Whether other gating checkpoints that contribute to the maintenance of the closed state also function as steric brakes or form interactions stabilizing the closed state is still obscure (Figure 6C). 

Furthermore, while the interaction sites between TM1 and the TM2/TM3 ring crucial for Orai1 gating were studied in detail, those between TM4 and the TM2/TM3 ring have not yet been characterized in depth (Figure 6D).

Lastly, the Orai family is composed of three isoforms, which possess in general similar activation mechanisms. Nevertheless, functional, structural, and pharmacological differences suggest isoform-specific distinct signal propagation and gating hotspots which need to be distilled in detail. The discovery of isoform-specific key determinants mediating Orai gating likely facilitates the development of drugs selective for a certain Orai isoform (Figure 6E). 

In addition to the detailed mechanistic dissections of the STIM1–Orai1 activation steps, the study of their coregulation further revealed novel insights in downstream signaling processes and disease-related signaling pathways. To obtain a more accurate and dynamic resolution of CRAC channel mechanisms, optogenetic engineering is highly suitable. Meanwhile, several studies [40,122,123,124] published the transfer of light sensitivity to STIM proteins using light-sensitive oligomerization domains, which were used to trigger downstream signaling processes and structure–function relationships. Light-sensitive Orai proteins acting independently of STIM1 are still highly awaited. Their activation could be triggered with high spatiotemporal control, independent of a series of STIM1 activation steps, while they might offer novel, dynamic insights into the Orai structure–function relationships and transpire as a suitable tool to study and govern downstream signaling processes.

Despite STIM1 and Orai1 being sufficient to establish CRAC channel activation, these molecular key players have been reported to interplay with a multitude of accessory proteins [125,126,127,128,129,130,131]. An in-depth understanding of their interplay will provide novel insights into native CRAC channel systems and potential new targets for fine-tuned impairment with pharmacological compounds. Among the currently available CRAC channel drugs, only a few possess higher, but not full, selectivity for Orai channels. Moreover, their sites of action are widely unknown [115,132,133]. Presently, available and ongoing characterizations of the structure–function relationship of CRAC channel components pave the way for the development of novel, selective drugs to treat, for instance, allergic disorders and autoimmune diseases. 

## Figures and Tables

**Table 2:** Double mutants each combining one GoF and one LoF mutations classified by their effect on constitutive activity.

**Inactive**		Mutations in					**Inactive**		Mutations in				
		N-Term	TM1	TM2	TM3	TM4			N-Term	TM1	TM2	TM3	TM4
**CETR LoF**	**MTR GoF**	**K85E**		L130S			**MTR LoF**	**MTR GoF**			H134W	V181K	
				H134A									A235C
				F136S									S239C
					V181K								P245L
					V181A						L138A	V181K	
					L185A	F250A						V181F	A235C
						S239C					H134A		
						P245L							A235W
				H134A									S239W
				I148S				**CETR**			H134W		_265_ANSGA_265_
				I148S	V181K								A235W
						P245L							_265_ANSGA_265_
				H134A									
				E149K			**Active**		Mutations in				
				E149K	V181K				N-Term	TM1	TM2	TM3	TM4
						P245L	**MTR LoF/*LoF^weak^***	**MTR GoF**		V102A	H134W		
				H134A	L174D						I148S		
						A235C					E149K		
						S239C						L174D	
						P245L						S179F	
					L174D	F250A					H134A	*L188S*	
					L185A							*V191N*	
				H134A	S179F							*L194S*	
				F136S									*M243S*
						A235C					H134A		
						S239C					*T142C*		
						P245L	**CETR LoF**		K85E	V102A			
	**CETR**	K85E				_265_ANSGA_265_					H134A		_262_AAA_264_
													_262_GGG_264_
													P245L
													_262_AAA_264_
													P245L
													_262_GGG_264_
													P245L
													L261D

The left table summarizes known inactive double mutants combining a CETR LoF with an MTR/CETR GoF. The upper right table shows inactive double mutants combining an MTR LoF with an MTR or CETR GoF. The lower right table summarizes active double mutants containing an MTR/CETR LoF with an MTR GoF.

**Table 3:** Classification of MTR and CETR LoF mutants according to their effects on an opening-permissive pore geometry and/or STIM1 coupling.

	Orai1 xx				
xx =	MTR LoF (e.g., H134W, S239W)	MTR LoF^weak^ (e.g., L188S, …)	K85E LoF	CETR LoF (e.g., E149K, L174D)	Hinge LoF (e.g., 3xG, 3xA)
**Activation via STIM1**	inactive				
**Activation via OASF L251S**	Slight activity	n.d.	inactive		
**Coupling to OASF L251S**	yes		reduced		
**Activity in the presence of an MTR GoF**	inactive	active	inactive		active
**TM1 cysteine crosslinking**	Corresponds to control conditions	n.d.	Corresponds to control conditions		n.d.
**Hydration profile**	Reduced number of water molecules	n.d.	Reduced number of water molecules		n.d.
**Activity in the presence of a MTR GoF and SS**	active	n.d.	inactive		n.d.
**Activity within a dimer (Orai1 LoF–Orai1 GoF)**	active	n.d.	inactive		n.d.
	**↓**	**↓**	**↓**	**↓**	**↓**
**Control of**	**pore geometry** (for activation by both STIM1 and GoF)	**pore geometry** (required for activation by STIM1 *but not a GoF*)	**pore geometry** (for activation by both STIM1 and GoF), **partly STIM1 coupling**	**pore geometry** (for activation by both STIM1 and GoF), **STIM1 coupling**	**pore geometry** (required for activation by STIM1 *but not a GoF*), **STIM1 coupling**

Orai1 LoF mutants can be grouped into MTR LoF, MTR LoF^weak^ (L188S, L194S, M243S), CETR LoF, and hinge LoF mutations. This division is based on their effects on STIM1-mediated activation, STIM1 coupling, an MTR GoF mutant, TM1 cysteine crosslinking, Orai1 hydration profile, activity in the presence of an MTR GoF, activity upon linkage to CAD–CAD fragments (-SS), and activity within a dimer, which are summarized in the table. The observed effects are leading to the conclusion as indicated by the arrow together with the last line. n.d., not determined.

## Data Availability

No new data were created or analyzed in this study. Data sharing is not applicable to this article.

## Abbreviations

2-APB	2-aminoethoxydiphenyl borate
Å	angstrom (unit of length equal to 10^−10^ m)
aa	amino acid
ANSGA	four-point mutation in hinge region aa position 261–265
Ca^2+^	calcium ion
CAD	Ca^2+^ release-activated Ca^2+^-activating domain
CAR	Ca^2+^-accumulating region
CC	coiled-coil
cCAD	*Caenorhabditis elegans* CAD
CETR	cytosolic extended transmembrane region
cOrai	*Caenorhabditis elegans* Orai
CRAC	Ca^2+^ release-activated Ca^2+^
cryo-EM	cryogenic electron microscopy
Cs^+^	cesium ion
C-term	C-terminus
cSTIM	*Caenorhabditis elegans* STIM
∆N	represents N terminal deletion mutants
dOrai	*Drosophila melanogaster* Orai
DVF	divalent-free
ER	endoplasmic reticulum
ETON	extended transmembrane Orai1 N-terminal
FCDI	fast calcium-dependent inactivation
FIRE	FRET-derived interaction in a restricted environment
FRAP	fluorescence recovery after photobleaching
FRET	fluorescence resonance energy transfer
GoF	gain of function
I_Ca^2+^_	CRAC current
I_Na+_	sodium current in sodium divalent-free solution
ID	inhibitory domain
IP_3_	inositol triphosphate
L-type	long-lasting calcium channel
LoF	loss of function
LV(SHK)	hinge region aa position 261–265
MD	molecular dynamics simulations
MTR	middle transmembrane region
Na^+^-DVF	sodium divalent free
nEF	noncanonical EF hand
NMR	nuclear magnetic resonance
N-term	N-terminus
OASF	Orai-activating small fragment
Orai 1–3	Orai proteins (also O1–3)
PBD	polybasic domain
PIP_2_	phosphatidylinositol 4,5-bisphosphate
PM	plasma membrane
SAM	sterile α-motif
S	signal peptide
SCDI	slow calcium-dependent inactivation
SOAR	*STIM*–Orai-activating region
SOCE	store-operated calcium entry
STIM	stromal interaction molecule
TAM	tubular aggregate myopathy
TM	transmembrane helices
TRP	transient receptor potential ion channel (C-canonical, M-melastatin, V-vallinoid)
